# Reconstruction of volume averaging effect‐free continuous photon beam profiles from discrete ionization chamber array measurements using a machine learning technique

**DOI:** 10.1002/acm2.13411

**Published:** 2021-09-06

**Authors:** Karl Mund, Luke Maloney, Bo Lu, Jian Wu, Jonathan Li, Chihray Liu, Guanghua Yan

**Affiliations:** ^1^ Department of Radiation Oncology University of Florida Gainesville Florida USA

**Keywords:** artificial neural network, ion chamber array, volume averaging effect

## Abstract

**Purpose:**

The use of the ionization chamber array ICProfiler (ICP) is limited by its relatively poor detector spatial resolution and the inherent volume averaging effect (VAE). The purpose of this work is to study the feasibility of reconstructing VAE‐free continuous photon beam profiles from ICP measurements with a machine learning technique.

**Methods:**

In‐ and cross‐plane photon beam profiles of a 6 MV beam from an Elekta linear accelerator, ranging from 2 × 2 to 10 × 10 cm^2^ at 1.5 cm, 5 cm, and 10 cm depth, were measured with an ICP. The discrete measurements were interpolated with a Makima method to obtain continuous beam profiles. Artificial neural networks (ANNs) were trained to restore the penumbra of the beam profiles. Plane‐specific (in‐ and cr‐plane) ANNs and a combined ANN were separately trained. The performance of the ANNs was evaluated using the penumbra width difference (PWD, the difference between the penumbra widths of the reconstructed and the reference profile). The plane‐specific and the combined ANNs were compared to study the feasibility of using a single ANN for both in‐ and cross‐plane.

**Results:**

The profiles reconstructed with all the ANNs had excellent agreement with the reference. For in‐plane, the ANNs reduced the PWD from 1.6 ± 0.7 mm at 1.5 cm depth to 0.1 ± 0.1 mm, from 1.8 ± 0.6 mm at 5.0 cm depth to 0.1 ± 0.1 mm, and from 2.4 ± 0.1 mm at 10.0 cm depth to 0.0 ± 0.0 mm; for cross‐plane, the ANNs reduced the PWD from 1.2 ± 0.4 mm at 1.5 cm depth, 1.2 ± 0.3 mm at 5.0 cm depth, and 1.6 ± 0.1 mm at 10.0 cm depth, to 0.1 ± 0.1 mm.

**Conclusions:**

This study demonstrated the feasibility of using simple ANNs to reconstruct VAE‐free continuous photon beam profiles from discrete ICP measurements. A combined ANN can restore the penumbra of in‐ and cross‐plane beam profiles of various fields at different depths.

## 1 INTRODUCTION

Accurate measurement of photon beam profiles is essential in the acceptance test of a linear accelerator (linac), the commissioning of a treatment planning system (TPS), and the periodic quality assurance (QA) of the linac.^1^, ^2^, ^3^ In standard practice, photon beam profiles are continuously scanned in a three‐dimensional (3D) water tank with detectors such as an ion chamber or a diode. The comprehensive data set collected after the acceptance test of the linac is used to commission the TPS beam model and serves as the baseline for periodic QA. In periodic QA, a smaller data set is collected with the 3D water tank and compared against the baseline to ensure that the linac performs within tolerance.

Due to the need of a large data set for TPS commissioning and periodic QA, water tank scanning can be very time consuming and inconvenient on some newly designed linac.^1^, ^4^, ^5^, ^6^ Both ion chambers and diodes are point detectors, meaning that the detector needs to translate across the entire field while the radiation is on. The detector translation speed is limited by the high demand of signal‐to‐noise ratio (SNR). Another issue with water tank scanning is that the quality of the beam data depends on the skills of the physicist performing the measurement. The results may vary depending on the scanning system and the choice of the detector. For example, ion chambers are the detectors of choice for scanning due to their wide availability and independence of beam energy and dose rate. However, ion chambers of large volume suffer from intrinsic volume averaging effect (VAE),^7^ while ion chambers of small volume have low SNR. Diodes are usually used in small fields to overcome the VAE of ionization chambers, but their response depends on beam energy and dose rate.^8^, ^9^ In some newly designed closed‐bore system such as the Halcyon linac (Varian Medical Systems, Palo Alto, CA), it is inconvenient to set up a conventional 3D water tank.

Recently, ionization chamber‐based detector arrays such as the ICProfiler (ICP, Sun Nuclear Corp., Melbourne, FL) have gained popularity due to their convenience and real‐time feedback.^5^, ^10^, ^11^ These arrays have discrete ionization chambers arranged on X and Y axes as well as the two diagonals. Simultaneous data acquisition over the entire open field is achieved while the beam is on, eliminating the need to translate a detector across the field. These ion chamber‐based detector arrays also offer independence of beam energy and dose rate. Although the ICP has great potential, it suffers from its poor spatial resolution and the intrinsic VAE of the ionization chambers. The detector spacing of the ICP is 5.0 mm along the X and Y axis and 7.1 mm on the diagonals, which is inadequate to sample the high gradient part of the beam profiles (penumbra). The individual ionization chambers have an active volume of 0.046 cm^3^ that contributes to intrinsic VAE. The VAE degrades beam data quality in the penumbra, which has significant clinical impact, especially for small fields.^7^

The purpose of this work was to investigate the feasibility of reconstructing continuous and VAE‐free photon beam profiles from ICP measurements with a machine learning approach. The use of machine learning technique in radiotherapy QA has become increasingly popular.^12^, ^13^, ^14^ The authors previously developed a machine learning‐based technique to eliminate the intrinsic VAE in ionization chamber‐measured photon beam profiles.^15^, ^16^ While our previous works addressed the VAE in continuous beam profiles, this work dealt with discrete beam profiles measured with a discrete detector array. To overcome the detector array's poor spatial resolution, each measurement was upsampled using the Makima interpolation. Then an artificial neural network (ANN) was trained to eliminate the intrinsic VAE and restore the high gradient penumbra. To evaluate the performance of the proposed technique, the reconstructed beam profiles were compared with those collected with a diode in a 3D water tank. We also compared the performance of plane‐specific ANNs (trained for in‐ or cross‐plane separately) against that of a combined ANN (trained for in‐ and cross‐plane combined) in the hope that a single ANN could be used for both axes of the ICP.

## 2 METHODS AND MATERIALS

**FIGURE 1:**
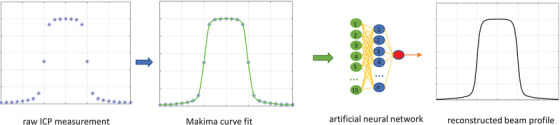
Workflow of the proposed method: the raw ICP measurement was interpolated with the Makima curve fit to obtain a continuous curve; the curve was input into a pretrained artificial neural network, which reconstructed a volume averaging free continuous beam profile. The reconstructed beam profile has quality similar to the beam profile scanned in a 3D water tank with a diode detector

Figure 1 outlines the workflow of the proposed method. Discrete beam profiles were collected with an ICP. Then the Makima curve fitting was used to upsample the beam profiles to obtain continuous profiles. These continuous profiles were used as input to the pretrained ANN that eliminated the intrinsic VAE and any effect caused by the ICP's poor spatial resolution. The output of the ANN was a continuous VAE‐free photon beam profile.

### 2.1 Data collection and preparation

In‐ and cross‐plane beam profiles of a 6 MV photon beam on an Elekta linac (Versa HD, Elekta Inc., Crawley, UK) were simultaneously measured with an ICP using the detectors on the Y and X axis, respectively. There are 65 detectors on the Y axis and 63 detectors on the X axis with a 5.0 mm spatial resolution, covering a 32 cm measurement length on both axes. On the X axis, there are no detectors at 5.0 mm from the center on either side due to space constraint. Fifty monitor units (MUs) were delivered for each measurement. The measurement geometry included seven field sizes (2 × 2, 3 × 3, 4 × 4, 5 × 5, 6 × 6, 8 × 8, 10 × 10 cm^2^) at three depths (*d*_max _= 1.5, 5, and 10 cm) for a total of 21 beam profiles in both in‐ and cross‐plane. The distance from the source to the surface of the ICP was 90 cm for all the measurements. The inherent buildup of the ICP is 0.94 g/cm^2^ and various thickness of solid water was added to achieve the desired effective measurement depths. The device has 2.3 g/cm^2^ inherent backscatter. Additional solid water of 10 cm was placed under the ICP to provide adequate backscatter. These measurements were repeated on three different days. The beam profiles were also collected with an EDGE diode detector in a 3D cylindrical water tank (Sun Nuclear Corp.). The EDGE diode has a 0.8 × 0.8 mm^2^ dimension and offers practically negligible VAE.^17^, ^18^ The diode‐measured beam profiles were used as reference to train the ANNs (see Section 2.3).

### 2.2 Neural network model

Motivation for this work stemmed from our previous work using an ANN to eliminate the VAE in ionization chamber‐measured photon beam profiles.^15^, ^16^ As the details of the ANN model can be found in previous papers, here we only outline its general structure. The three‐layer ANN consists of an input layer, a hidden layer, and an output layer (Figure 1). While the input layer and hidden layer contain multiple nodes, the output layer has a single node. A sliding window (SW) was used to extract data points from the input profile to serve as the input to the ANN. The ANN outputs the value representing the reconstructed profile at the center of the SW. The relationship can be illustrated by the following equation:

(1) $$O\;=\sigma_{o}\;\left(\sum_{k\;=\;1}^{N_{hn}}w_{k}^{o}\sigma_{h}\left(\sum_{j\;=\;1}^{L_{sw}}w_{jk}^{h}s_{j}+b_{k}^{h}\right)+b^{o}\right)$$

where *O* is the single‐valued output. $\sigma_{h}$ and $\sigma_{o}$ are the activation functions in the hidden layer and the output layer, respectively. In this work, the hidden layer uses the tangent sigmoid function and the output layer uses the linear activation function. $w^{h}$ and $w^{o}$ are the weights connecting the input layer with the hidden layer, and the hidden layer with the output layer, respectively. $b^{h}$and $b^{o}$ are the biases. $N_{hn}$ and $L_{sw}\;$denote the number of hidden nodes and the length of the sliding window, respectively. Our previous experience shows that a sliding window covering 1.5 cm length works well for all the studied geometries.^16^ The value of $N_{hn}$ may depend on the specific problem. In this work, the optimal $N_{hn}$ was determined via a parametric sweeping method (Section 2.3).

### 2.3 Network training

On Elekta Versa HD linacs, the in‐plane field edge is defined by a pair of collimator jaws situated lower than the multi‐leaf collimators that define the cross‐plane field edge. As a result, the in‐plane beam profiles have sharper penumbra than the cross‐plane beam profiles. In our data collection, we noticed that the difference in the penumbra between the ICP measurements and the diode scans was larger in the in‐plane direction than in the cross‐plane direction. This prompted us to first train plane‐specific ANNs: one ANN for in‐plane only (in‐plane ANN) and another one for cross‐plane only (cr‐plane ANN). Then a combined ANN was trained using in‐ and cross‐plane beam profiles combined. The performance of the plane‐specific ANNs was compared against that of the combined ANN to determine the feasibility of using a single ANN for both planes.

The beam profiles of the 2 × 2, 4 × 4, 6 × 6, and 10 × 10 cm^2^ fields at all three depths were used to train the ANNs. A total of 16 416 data pairs (input and reference output) were extracted from these profiles. The input was extracted with the sliding window and the reference output was extracted from the diode‐measured beam profiles (ground truth). These data pairs were divided into training (70%), validation (15%), and test (15%) dataset to train the ANNs. The beam profiles of the 3 × 3, 5 × 5, and 8 × 8 cm^2^ fields were used to evaluate the performance of the ANNs. The beam profiles from respective planes were used to train the plane‐specific ANNs. The beam profiles from both planes combined were used to train the combined ANN.

The optimal $N_{hn}\;$of the plane‐specific and the combined ANNs was separately determined using a parametric sweeping method. Each ANN was trained with $N_{hn}\;$varied from 2 to 20 (with a step size of 2). The ANNs were initialized with random weights and biases to prevent entrapment in local minima. The Levenberg–Marquardt backpropagation algorithm was used to update the weights over 400 epochs of training. For each $N_{hn}$, the training was repeated 10 times and the one that minimized the mean squared error (MSE),

(2) $$\mathrm{MSE}\;=\;\frac{1}{N}\sum_{i\;=\;1}^{N}{\left(O_{i}-P_{i}\right)}^{2},$$

between the predicted output, $O_{i}$, and the reference value, $P_{i}$, was recorded. *N* is the length of the profile. The ANNs with the $N_{hn}\;$that yielded overall minimal MSE were selected in the following evaluation.

The performance of the ANNs was evaluated with the penumbra width difference (PWD). The penumbra width was defined as the distance between the 20% and 80% intensities of a beam profile and the PWD was defined as the difference of the penumbra width between the reconstructed and the reference profiles, calculated using the following formula:

(3) $$\mathrm{PWD}\;=\;\left|W_{o}-W_{r}\right|$$

where $W_{o}$ is the penumbra width of the reconstructed profile and $W_{r}$ is the penumbra width of the reference profile. Smaller PWD indicates better agreement in the profile's high gradient region. Gamma analysis with 1%/1 mm criterion was also used to quantify the agreement between the reconstructed beam profiles and the reference profiles.

## 3 RESULTS

**FIGURE 2:**
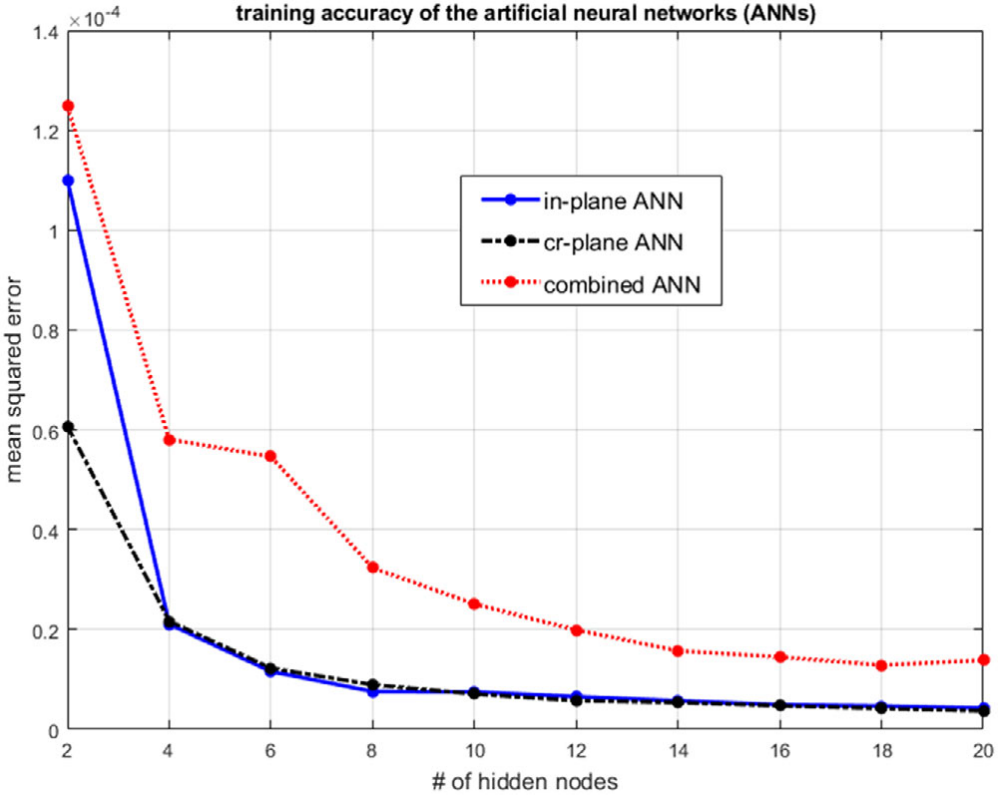
Training accuracy of the artificial neural network (ANN) as a function of the number of hidden nodes. Three ANNs were trained: one for in‐plane profiles only (“in‐plane ANN”), one for cross‐plane profiles only (“cr‐plane ANN”), and one for in‐ and cross‐plane profiles combined (“combined ANN”). Each ANN was trained 10 times with different initial weights and the minimal mean squared error was used in the plot. [Correction added on Sep 19, 2021 after first online publication: the figure caption is updated.]

Figure 2 shows the training accuracy of the ANNs as a function of the number of hidden nodes. In general, the MSE of all three ANNs decreased when the number of hidden nodes increased. The two plane‐specific ANNs achieved similar accuracy when $N_{hn}\;$≥ 4. The combined ANN performed slightly worse than the plane‐specific ANNs. Based on the results in Figure 2, the number of hidden nodes chosen for all three ANNs was 18. The ANNs were easy to set up and quick to train. The ANNs were implemented with Matlab (Mathworks, Natick, MA). The training of all three ANNs was completed in less than 3 min without the use of a graphics processing unit.

**FIGURE 3:**
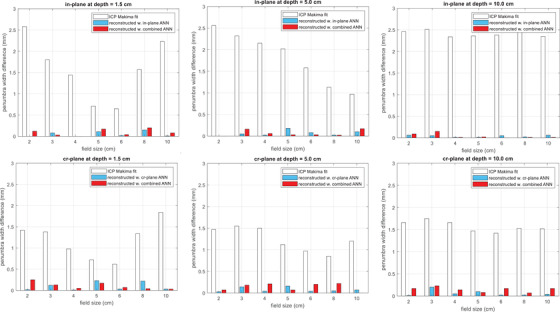
Performance of the plane‐specific artificial neural networks (ANNs) and the combined ANN evaluated with penumbra width difference (PWD) with respect to reference beam profiles. The in‐plane, cr‐plane, and combined ANNs all significantly reduced the PWDs of the ICP measurement interpolated with Makima method. Note the profiles from the 2 × 2, 4 × 4, 6 × 6, and 10 × 10 cm^2^ were used in the training; the ones from the 3 × 3, 5 × 5, and 8 × 8 cm^2^ were used for test. [Correction added on Sep 19, 2021 after first online publication: the figure caption is updated.]

Figure 3 shows the PWDs of the raw ICP measurements with the Makima interpolation and the beam profiles reconstructed with the plane‐specific ANNs and the combined ANN for individual fields. Table 1 shows the mean PWD at each depth averaged over all the field sizes using day one's measurements. For the Makima‐interpolated ICP measurements, the in‐plane showed larger PWDs than the cross‐plane in general; larger PWDs with less variations were observed at 10 cm depth than at the other two depths. The maximum mean PWD of 2.4 ± 0.1 mm was found in the in‐plane at 10 cm depth. The two plane‐specific and the combined ANNs all significantly reduced the PWDs after reconstruction. At all three depths, the profiles reconstructed with each ANN had mean PWD under 0.1 ± 0.1 mm in both in‐ and cross‐plane. In general, the plane‐specific ANNs performed slightly better than the combined ANN with smaller PWDs. However, after reconstruction, the largest PWD was 0.24 mm for the plane‐specific ANNs (cross‐plane of 5 × 5 cm^2^ at 1.5 cm depth) and 0.25 mm for the combined ANN (cross‐plane of 2 × 2 cm^2^ at 1.5 cm depth). Therefore, the performance difference between the plane‐specific ANNs and the combined ANN was practically negligible. The performance of all the ANNs was nearly identical on the data collected on three separate days with mean PWD being no more than 0.1 ± 0.1 mm. Note the profiles from the 2 × 2, 4 × 4, 6 × 6, and 10 × 10 cm^2^ were used in the training; the ones from the 3 × 3, 5 × 5, and 8 × 8 cm^2^ were used for the test. The performance of the ANNs on the training and test data set was nearly identical. The gamma pass rate with a 1%/1 mm criterion was 100% for all the studied geometries with all three ANNs.

**TABLE 1:** Penumbra width difference (PWD) of the ICP measurements with Makima fit, and the beam profiles reconstructed with the plane‐specific and the combined artificial neural networks (ANNs). All the ANNs achieved similar results on the training and test data sets. The standard deviation of all the PWDs was ≤0.1 mm. The maximum PWD was 0.25 mm (combined ANN, cross‐plane of 2 × 2 cm^2^ at 1.5 cm depth)

	in‐plane PWD (mm)			cr‐plane PWD (mm)		
		Reconstructed			Reconstructed	
Depth	ICP (Makima fit)	in‐plane ANN	Combined ANN	ICP (Makima fit)	cr‐plane ANN	Combined ANN
1.5 cm	1.6 ± 0.7	0.1 ± 0.1	0.1 ± 0.1	1.2 ± 0.4	0.1 ± 0.1	0.1 ± 0.1
5 cm	1.8 ± 0.6	0.1 ± 0.1	0.1 ± 0.1	1.2 ± 0.3	0.1 ± 0.1	0.1 ± 0.1
10 cm	2.4 ± 0.1	0.0 ± 0.0	0.0 ± 0.1	1.6 ± 0.1	0.1 ± 0.1	0.1 ± 0.1

**FIGURE 4:**
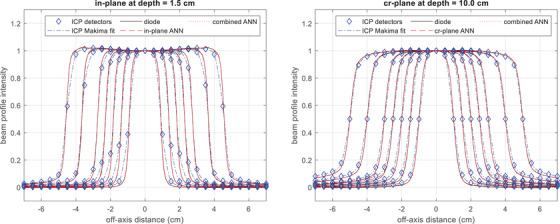
Examples of discrete ICP measurements along with Makima curve fit, and the beam profiles reconstructed with plane‐specific artificial neural networks (ANNs) (in‐plane or cr‐plane ANN) and the combined ANN. The profiles reconstructed by all three ANNs overlap the diode‐measurement profiles, which are considered the ground truth in this work. Note the profiles from the 2 × 2, 4 × 4, 6 × 6, and 10 × 10 cm^2^ were used in the training; the ones from the 3 × 3, 5 × 5, and 8 × 8 cm^2^ were used for test. [Correction added on Sep 19, 2021 after first online publication: the figure caption is updated.]

Figure 4 shows examples of the ICP measurements along with Makima interpolations, and the beam profiles reconstructed by the plane‐specific and the combined ANNs. For each beam profile, the reconstruction took less than 1 s. Again, larger deviation in the penumbra of the ICP measurement was observed in the in‐plane than in the cross‐plane. In each field geometry, the beam profiles reconstructed by the plane‐specific ANN and the combined ANN were almost identical, and both overlapped the diode‐measured profile. These results show that the combined ANN was equally effective as the plane‐specific ANNs in restoring the penumbra of the ICP measurements.

## 4 DISCUSSION

The use of ICP is limited by its poor spatial resolution and the VAE associated with the ionization chambers. The motivation of this work was to address these issues by leveraging the power of machine learning techniques. This study attempted to answer three specific questions in reconstructing continuous beam profiles from the ICP measurements: (1) Is it feasible to overcome the insufficient detector spatial resolution? (2) Can machine learning techniques such as the ANN eliminate the inherent VAE? (3) Can a single ANN work for both in‐ and cross‐plane beam profiles? Our results show that it is feasible to reconstruct VAE‐free continuous beam profiles at various field sizes and depths from discrete measurements with a single ANN.

There are two main factors that contribute to the difference in the penumbra of beam profiles measured with the ICP and the EDGE diode: the ICP's insufficient detector spatial resolution and the VAE associated with the ionization chambers. The Makima interpolation was used to convert discrete measurements into continuous beam profiles. It produces undulations that find a nice middle ground between piecewise cubic Hermite interpolation (PCHIP) and spline curve fit. It also eliminates overshoot associated with typical spline curve fit on photon beam profiles. The examples in Figure 4 show that Makima interpolation works very well, preserving the shape of the beam profiles from small to large fields at various depths. The influence of the VAE on the PWD can be understood by referring to a commonly used scanning ionization chamber CC04 (Scanditronix Wellhofer, Bartlett, TN). The ionization chambers of the ICP and the CC04 have similar volumes (0.046 cm^3^ vs. 0.040 cm^3^), but their dimension along the field gradient direction is smaller than that of CC04 (2.9 mm vs. 4.0 mm). Therefore, the ICP is expected to have less VAE than CC04, which is in line with our observation in previous experiments.^17^ The typical PWD observed on measurements with the CC04 is 2.0 mm,^17^ while we found that the mean PWD of the ICP measurements was under 2.0 mm except for the in‐plane at 10 cm depth where the mean PWD was 2.4 ± 0.1 mm. This exception may be partially attributed to the effect of the Makima interpolation, which depends on where in the penumbra that the individual detectors sample the profile. Note that additional measurements by shifting the detector could be used to further improve data density and reduce the impact of the spatial resolution, but significantly more work would be added, which becomes impractical when a large amount of profiles need to be collected. Karimnia et al. used the ICP to collect the beam profile of a 2 × 2 cm^2^ field up to 10 times by shifting the detector 0.5 mm in between.^10^ Then the gamma comparison was used to compare the combined beam profile with the diode‐measured beam profile (the diode‐measured beam profile was convolved with the detector's response function). The 1%/1 mm gamma passing rate was merely 65.7%. Their results suggest that increasing detector spatial resolution by shifting the ICP and taking multiple measurements cannot eliminate the difference in penumbra. A deconvolution method is warranted to restore the high gradient penumbra. In this work, we found that the ANN is a suitable technique for such purpose. Combined with a Makima interpolation, the ANNs not only made up for the insufficient detector spatial resolution, but also eliminated the intrinsic VAE in the ICP.

There are a few limitations in this study. We only included beam profiles of fields no larger than 10 × 10 cm^2^ at three depths. Highly conformal radiotherapy mainly uses small beam segments to paint the dose. The impact of the VAE is relatively more important in these applications. If desired, the proposed technique can be extended to larger fields since the ICP has a maximum field size of 32 × 32 cm^2^. Additionally, reduced source‐to‐surface distance can be used to cover the entire 40 × 40 cm^2^ field. On the other hand, the difference in the scattering condition in large fields (lack of full scatter due to the limited size of the ICP) comes into play and needs special consideration. As far as the depth is concerned, our linac is adjusted to produce relatively flat beam profiles at 10 cm depth with a flattening filter. As a result, the beam profiles show “horn” effect at shallower depths and “shoulder” effect at deeper depths. However, the magnitude of such effects is small in fields ≤10 × 10 cm^2^. Figure 4 shows that the ANN can preserve the horn effect learned from the training data very well. It is expected to also preserve the shoulder effect at deeper depth. Additionally, a diode was used to collect a reference data set for the ANN, which tends to overrespond to low energy scattering photons. This is another reason that we limited our data set to field sizes below 10 × 10 cm^2^. Another limitation of this work is that the dosimetric impact caused by the density difference between the ICP and homogenous water was not explicitly addressed. It appears that the ANN implicitly accounts for the difference in the training. Another point worth noting is that we assumed neligible VAE in the EDGE diode. With a 0.8 × 0.8 mm^2^ dimension, the EDGE diode has been used as a reference detector in previous studies.^17^

The feasibility of reconstructing VAE‐free continuous photon beam profiles from discrete ICP measurements was proved in this study, but more research needs to be done for this technique to be clinically useful. The proposed technique needs to be validated at various beam geometries or clinical settings (e.g., field size, depth, source‐to‐surface distance, linacs of the same model, and linacs of different models). A machine learning model independent of these variables would be most useful in practice. In that case, the vendor could provide device‐specific machine learning models, thereby eliminating the requirement on the end users to train the model. The proposed technique can be especially useful in some newly advanced linac, where the vendor supplies a golden beam data set and a TPS model, and the purpose of the user data collection is to validate that the linac produces beam profiles that match the golden beam data set.

## 5 CONCLUSION

In this work, we demonstrated the feasibility of reconstructing VAE‐free continuous photon beam profiles from ICP measurements using a simple machine learning technique. Combined with Makima interpolation, the three‐layer ANNs can not only make up for the device's insufficient spatial resolution but also eliminate the intrinsic VAE. We also showed that a single ANN can be trained for both in‐ and cross‐plane. Further work is needed to study whether the same ANN model can be applied to beam profiles collected with different source‐to‐surface distances, beam energies, and different linacs.

## AUTHOR CONTRIBUTIONS

Karl Mund, Luke Maloney, and Guanghua Yan collected all the data; all authors participated in the method design; Karl Mund and Guanghua Yan drafted the manuscript; all authors reviewed the manuscript.

## CONFLICT OF INTEREST

The authors declare no conflict of interest.
